# Supplementary material for: Data-driven study of the COVID-19 pandemic via age-structured modelling and prediction of the health system failure in Brazil amid diverse intervention strategies
Source: PLoS One. 2020 Jul 30;15(7):e0236310. doi: 10.1371/journal.pone.0236310 (PMC7392258; doi:10.1371/journal.pone.0236310)
Supplement: Attachment — Submitted filename: Response to Reviewers.pdf [file pone.0236310.s001.pdf]

June 9, 2020

To the Editor of PLOS ONE -

Dear Lidia Adriana Braunstein,

Thank you for your correspondence on our manuscript PONE-D-20-09888. We also thank the Referees for the careful reading of the manuscript and for the relevant questions and comments. Hereby we address in details all comments and question made by the Referees.

Referee A does not recommend publication for the present manuscript. In the reply, we counterargument many of the criticisms of the Referee (using references and data).

Referee B recommends publication saying that the paper "The paper is well written and clear and I recommend that the manuscript be published. However, I have a few comments that I believe may improve the ms (manuscript).".

Referee C recommends publication saying that the paper "The manuscript is technically solid. The data used in the analysis is available at Johns Hopkins University COVID-19 database. The method used is well known and has been used in similar articles".

Overall, the majority of the reports reaffirms the feedback we have obtained from the scientific community so far and strength our understanding that those kind of papers represent an important interdisciplinary contribution for COVID-19 as well as new coming global-scale viral diseases. The interdisciplinary aspect of the paper is surely adherent to the broad PLOS ONE audience.

Below we a make a point-by-point answer to the Referees, also highlighting the specific changes made in the manuscript.

With that we hope our manuscript can now be considered ready for publication in PLOS ONE.

Sincerely,

Askery Canabarro, on behalf of all the authors.

1) Additional Requirements

- We followed PLOS ONE's style requirements template and we believe it is in agreement with the guidelines;
- We declare the data sets are all from open sources. It can be found in famous COVID-19 repositories such as John Hopkings University `https://coronavirus.jhu.edu/`;
- Funding Statement should read: "John Templeton Foundation via the Grant Q-CAUSAL No. 61084, the Serrapilheira Institute (Grant No. Serra-1708-15763), the Brazilian National Council for Scienti c and Technological Development (CNPq) via the National Institute for Science and Technology on Quantum Information (INCT-IQ) and Grants No. 307172/2017-1 and No. 406574/2018-9 and No 423713/2016-7";
- Acknowledgments now reads only: "We thank the Brazilian agencies MCTIC and MEC. AC also acknowledges UFAL for a paid license for scienti c cooperation at UFRN";
- We referred to all Tables in the main text (including Table IV).

2) List of changes

- We revised the entire manuscript, searching for typos;
- We have corrected all the captions and titles in Tables and Figures, specially Table IV;
- We better discuss the results of Table V and VI as suggested by the referees, adding a sentence saying that their estimations is based from the initial date up to 150 days ahead, where the disease is modelled to be controlled;
- We have added a sentence saying the "In fact, the variables are fractions of the respective compartments, i. e., $s' = S/N$, $i' = I/N$, $r' = R/N$ and $d' = D/N$", just after the introduction of the compartments and population size N as suggested by the referees;
- We have expanded the axes limits in Fig. 2 so that it can be easily inferred the performance in the short term.

- All changes are highlighted in bold face in one version, as recommended.

Reply to the Referee A

We thank the referee for the careful and thoughtful reading of our paper. In the new version we have taken all comments into account and we believe that now the paper delivers its message in a clearer way.

Referee A: *Reviewer 1: The work "Data-Driven Study of the COVID-19 Pandemic via Age-Structured Modelling and Prediction of the Health System Failure in Brazil amid Diverse Intervention Strategies" presents a model built by coupling SIR models for different ages, thus introducing an age structure. The true variables of the model are fractions of populations (s=S/N, r=R/N,...) where N is the population of Brazil, more than 200 million people. The age-populations are coupled by a matrix of contagious contacts which originally is a multiple of matrix with all ones, 1, in its entries, but later is modified to accommodate what the authors speculate is the translation into parameters of the non pharmaceutical interventions (NPI).*

Our reply: We have inserted a sentence after the model description stating that, in fact, the variables are fractions of populations as the referee correctly pointed out. Also, as we have mentioned openly in our manuscript, the contact matrix is a limitation in our model with 9 sub-populations. At the moment of writing (and still today due to the immense under-reporting in Brazil) there is no solid foundation for how we could describe or retrieve this contact matrix in a more detailed manner. That is precisely the reason why we have opted for a data-driven study, in order to minimize any number of assumptions without clear reasoning.

Therefore, we decided that it would be a topic for further investigation, potentially by means of complex network theory, in similar lines as done, for instance, in PRX 10, 011070 (2020) and PRL 124, 068301 (2020), modelling human contact networks using variants of networks topologies. We discuss more ahead.

Referee A: *There is no support in the biomedical literature for the original (before interventions) contact matrix, actually mild cases are expected to be less contagious than severe cases (at e very primitive level, think that a good number of mild cases do not present cough, one of the predominant mechanisms for contagious). The immunological system is also expected to wear off with age in average. Thus, the contagious contact matrix the authors use as a first step is not supported by present knowledge of SARS-CoV-2. The same can be said for recovery times.*

Our reply: The point made by the Referee is relevant but we understand that our manuscript deals with those comments in a variety of ways. More specifically.

- Independent works have been done specifically to investigate the contact matrix within some communities, such as [PLOS Computational Biology 13, 1 (2017)], which we properly cite in the new version of the manuscript. However, this demographic fingerprint of a specific group would be not directly and easily transposed to other communities, as one can deduce from Fig. 1 extracted from the reference mentioned above. Notice how distinct the patterns are for distinct countries. We selected only two locations (home and work), more can be found in the paper.

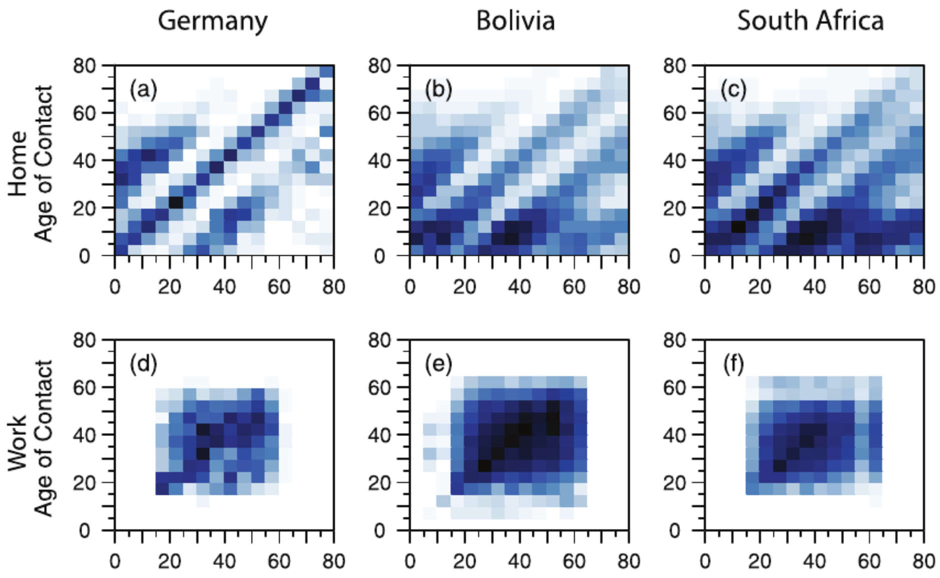

FIG. 1: Contact matrix for distinct countries and locations.

Although an uniform contact matrix is a crude first approximation, a more detailed description can be incorporated in the infection rate parameter β (that is age structured in our model) as in $(\beta^{'}C)SI$, explaining why communities with intense contact show a fast contagious rate (larger β). In other words, given the difficulty in describing the contact matrix, we have made a conscious choice to neglect it and rely solely on data-driven estimation of the infections rate as an effective measurement of the joint effects (probability of infections when exposed and the contact matrix). From Fig. 1, we note that is not absurd to consider a uniform matrix as first approach. However, for sure, to be more realistic we would need to have such work done specifically for Brazil and clearly this is beyond the scope of this work.

- In the absence of vaccines, preventive medicines as well as any effective established protocol to treat patients, overall the virus faces a battle against the human immune system. This is precisely what our model is doing, we neglect any pharmaceutical interventions. We do not need to insert in the model any potential immune system aging effect once it is incorporated in the data-driven recovered and death rates per age α and γ, respectively. The explanation of increasing rates in function of age can be either the wear off of the immune system or the higher incidence of comorbidities such as obesity, cardiac pathologies etc, as already discussed in the main text. This is beyond the scope of an epidemic modelling as we investigate the spreading of the disease from host to host, not the propagation of the virus within the host.
- The recovering time can be retrieved directly from the recovery rate, one being the inverse of the other. Again, it is implied from data and is not an arbitrary choice. The explanation for longer recovery times for older people follow the same previous explanation.
- We would like to stress that our team of researchers in this project was composed of three physicists and three physicians and we did our best to keep track of the most relevant evidences about SARS-CoV-2 available at the time of writing, around April 3, 2020. The ground truth is that, so far, we still don't know much about the full contagious variables and we behave more or less the same way we would do centuries ago (Black Death times) during a pandemic disease: i) applying social distance, ii) together with the gained secular knowledge that hygiene is also a powerful tool. To the best of our knowledge the claim of the Referee that "think that a good number of mild cases do not present cough, one of the predominant mechanisms for contagious" has no scientific background or consensus.
- Below we show some results on the contagiousness of COVID-19 and the respective references. It is possible to observe that mild cases are not negligible carriers as conjectured by the referee. In fact, together with asymptomatic persons (less likely to transmit the virus, `WHO`) they can potentially be the main spreaders as these individuals can be freely moving in comparison with people with severe symptoms and/or in hospitals. So, our approach of not separating the infected compartment into classes (asymptomatic, mild/moderate, severe and critical) seems to have scientific background, at least for a first approximation as by not separating one is assuming the infectiousness of a given person to be independent of the intensity of the symptoms to be shown, i. e., lower contagiousness roughly being compensated with higher mobility. This can be easily inferred from Fig. 2, which is a schematic representation of the results found in [Ann. Intern. Med., 172,577-582, 2020], `https://www.acpjournals.org/doi/10.7326/M20-0504`.

Fig. 2 also suggests that most people who become infected may not even know they are infectious. It leads to the assumption that everyone is a potential pre-asymtomatic and can become contagious unless negatively tested for the virus or antibodies to the virus, since people appear to be infectious for the first four days before experiencing symptoms (minor contribution) and during the symptomatic stage (major contribution), however it is impossible to assert *a priori* if someone will or not present symptoms. It seems to be a remarkable difference between COVID-19 and the disease related with SARS-Cov in 2002, where the removal of just symptomatic individuals was an effective control measure. This has proven not to be the case for COVID-19, given its wildfire-like spread evne under distinct control measures.

Referee A: *The authors leave aside that the SIR model does not represent a proper progression of contagiousness. Furthermore, the homogeneous mixing implicitly assumed of the population is known to be a problematic assumption to results in exaggerated number of contagious. In turn, the reduction factors associated to the NPI are rather arbitrary and more likely than over-optimistic. The effectiveness of a quarantine is related to the social structure and is going to be rather heterogeneous. It is almost impossible to prevent contact to families living together and is almost impossible to restrain to their home to those which leave under precarious conditions. I am thinking of shanty towns (Favelas), Comunitary isolation is more likely to happen in such social conditions. As a consequence, results such as those in Figure 3 are truly fabulated. I fail to see a reason to recommend to PLOS' readership the present manuscript.*

Our reply:
- Overall, the referee seams skeptical about the application of SIR-like models to acquire useful insights about human-human diseases propagation. We respect this point of view of the Referee, however it is one of the most used paradigm of epidemic modelling. In fact, as can be inferred from the report, usual simplifications are not well digested by the referee. For instance, the common uniform contact matrix, which has been used since its creation in 1927 for predicting the number and distribution of cases of an infectious disease as it is transmitted through a population over time.

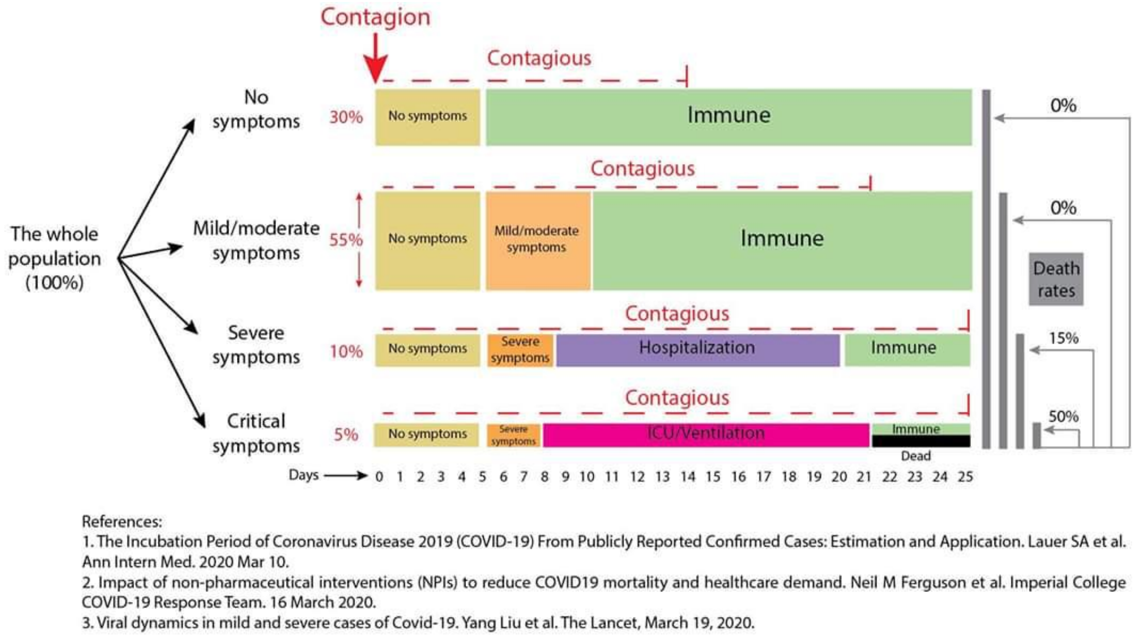

FIG. 2: Incubation and inctious period of COVID-19.

- Although very simple, the SIR model is proven to retrieve the same results of more advanced models such as epidemic processes in complex networks, for instance, see “Rev. Mod. Phys. 87, 925 (2015) - Epidemic processes in complex networks”. These models simulate the spread of the disease from one person to another taking into account the existence of a direct connection between them. That approach has already been used to study the COVID-19 (see “SOCIAL INTERACTION LAYERS IN COMPLEX NETWORKS FOR THE DYNAMICAL EPIDEMIC MODELING OF COVID-19 IN BRAZIL” arxiv:2005.08125)

- Models are always simplifications, which can lead to the proposition that they are intrinsically wrong, but that does not mean that models are not useful, specially for short-range predictions or for qualitatively compare different scenarios as we do in our paper by considering differente NPIs.

- As shown in Fig. 2 of the manuscript, we used only data up to 21th March 2020, validated with data up to 1st April 2020 and (not shown in the first version of the paper, but now partially inserted) it remained a good estimator up to around 10th April 2020, without the necessity of any re-calibration, meaning it could perform well for around 3 weeks for the number of deaths. To have a good performance beyond this time frame one can adapt our model and consider for instance the variability in time of the social distancing and reproduction rate R_0. In particular, the red curve is a created ”going back in time” as explained in the main text, therefore it is clearly speculatory, just to show the theoretical effects of NPIs polices over the disease propagation. All these limitations we openly pointed out in our manuscript.

- Furthermore, the NPIs policies we modelled are not arbitrary, once for every measure we give an estimation of the effect if it is fully respected. For instance, if the measure is closure of schools, we provide the percentage of the population affected by using reliable and up to date census data. The only assumption is the compliance by the population, which we limited up to 75% excepted for the obvious ones (closure of schools and universities). In fact, the Brazilian’s current social distance ratio modelled to be 0.5 could be retrieved from the Google’s Community Mobility tool, available at `https://www.google.com/covid19/mobility/`.

- The main message of the paper is how to protect the health system, once we found that it would start to collapse in Brazil within a short-term range (the time frame which the model can give quantitatively good predictions). Unfortunately, this was exactly what happened as our prediction that the health system would start to collapse around the end of April 2020 materialized in many states of Brazil (most noticiably in Amazonas, Pará and Ceará). Currently, Brazil has more than 700.000 confirmed cases (estimated to be actually over 5 million) and current predictions by the John Hopkins University estimated around 130 thousand deaths by the beginning of August 2020. At the time of writing, the long term predictions in Tables V and VI by 1st April (being long term, are only qualitative) seemed impossible. Unfortunately, they seem very plausible at the moment.

- Finally, we must say that SIR like models are a well-established paradigm allowing for good quantitive short term

predictions (as unfortunately, it is the case with our results) and overall qualitative comparison predictions in the long term. This tool is being used by many governments and in particular in Brazil it is a central tool by many local health offices planning the opening/closing of activities. As a matter of fact, after the pre-print appeared, some of us start to collaborate actively in this urgent endeavours. We respect the referee's position against this kind of modelling but to neglect a widely used approach (on which there is consensus in favor of its short time range predictions) seems rather arbitrary and unfair. Please, check also the following replies for more information.

Reply to the Referee B

We thank the Referee for the positive assessment of our paper and for recommending publication. We have adressed all the modifications suggested by the Referee, as detailed below.

Referee B: *Reviewer 2: In this work the authors propose a data-driven age-structured census-based SIRD-like epidemiological model capable of forecasting the spread of COVID-19 in Brazil. They model the current scenario of closed schools and universities, social distancing of individuals above sixty years old and voluntary home quarantine, and show that it led to a considerable reduction in the number of infections as compared with a scenario without any control measures. However, the authors predicts that the current measures are not enough to avoid overloading the health system, since the demand for intensive care units will soon surpass the number available and that an urgent intense quarantine might be the only solution to avoid this scenario and, consequently, minimize the number of severe cases and deaths.*

The paper is well written and clear and I recommend that the manuscript be published. However, I have a few comments that I believe may improve the ms.

Our reply: We thank the Referee again for the positive report. Below we provide our replies.

Referee B: *1. In page 4, after the reproduction number* R_0 *is definited, the same magnitud is named as* R_n*. At least I have missing something I think the authors are talking about the same magnitud.*

Our reply: We thank the Referee for the careful reading. However, the difference was intentional once R_0 is the term for the very first beginning of the disease, where $S \approx N$. Now, for instance, with more than half million of confirmed cases (likely around $5M$ cases in reality) and ten of thousands of deaths, we prefer to use this different notation. A quick explanation is the following taking a simple SIR model as a benchmark.

$$\frac{dS(t)}{dt} = -\beta \frac{S}{N} I, \tag{1}$$
$$\frac{dI(t)}{dt} = \beta \frac{S}{N} I - \gamma I, \tag{2}$$
$$\frac{dR(t)}{dt} = \gamma I. \tag{3}$$

If the initial assumption $S \approx N$ holds, therefore $S/N \approx 1$, yielding

$$\frac{dS(t)}{dt} = -\beta I, \tag{4}$$
$$\frac{dI(t)}{dt} = \beta I - \gamma I, \tag{5}$$
$$\frac{dR(t)}{dt} = \gamma I. \tag{6}$$

So, Eq. (5) would have the solution $I(t) = e^{(\beta/\gamma)t}$, and yield the definition of $R_0 = \beta/\gamma$. If Eq (5) had one more term, for instance, $-\alpha I$, as in our model, $R_0 = \beta/(\gamma + \alpha)$. One can show that in the very beginning (it holds for few days), R_0 can crudely be approximated as number of new infected persons due to a given infectious individual (geometric progression with common ratio R_0). Apart from this very initial period, one should avoid keeping the same terminology, hence why we used R_n for later evolution stages. However, we must notice, it is very common in the literature to keep the same notation (note that in Fig. 3, only to appear in this Reply, we have done that) .

Referee B: *2. In page 7, line 3 from the bottom, the phrase "The third column of Table II is the unit minus the values in the third column of Table I" it is wrong. I think that the third column of Table II is the unit minus the values in the fourth column of Table I. Please check this.*

Our reply: It is right. We have modified it.

Referee B: *3. Coronavirus report given by the World Health Organization (WHO) indicates that the total number of infected in Brasil by May 1st is of 78162 confirmed cases, less than* 10^5 *("https://www.who.int/emergencies/diseases/novel-coronavirus-2019/situation-reports"). This value is very different from the one estimated by the model (showed in Fig. 3a), which is over the* 10^6 *cases. Can the authors explain this huge difference?*

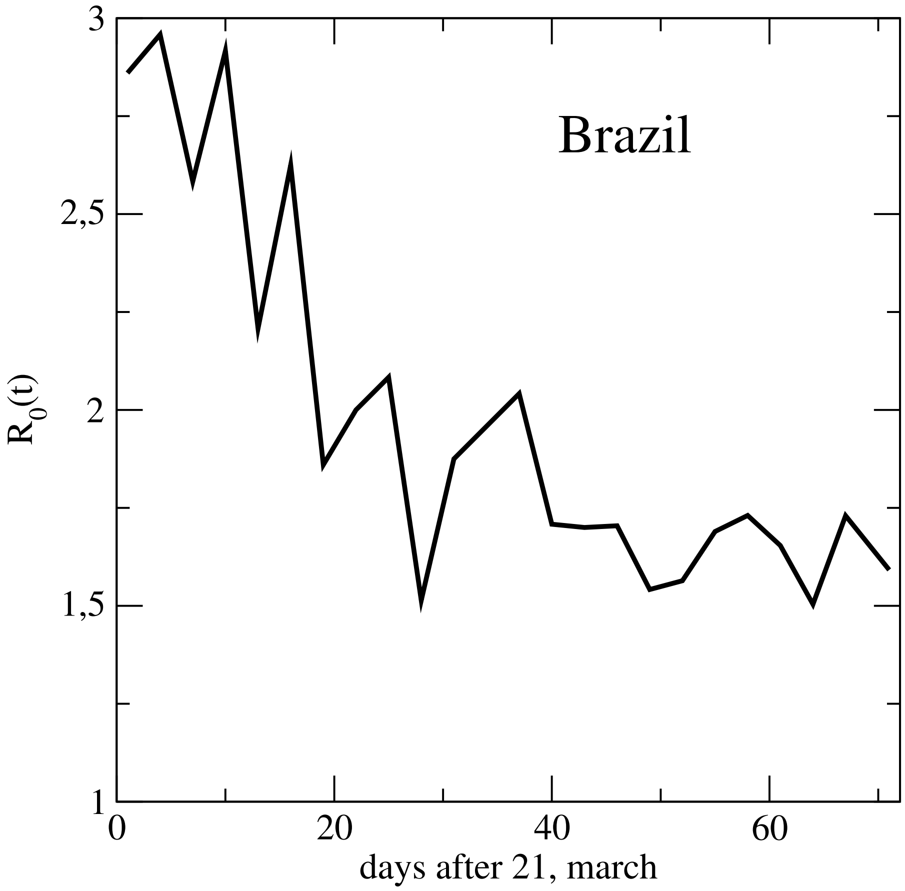

FIG. 3: R_0 as a function of time measured with best fit as in main text.

Our reply: This is in fact a very interesting and relevant point. We have two main explanations for this difference between the prediction and the confirmed cases by the beginning of May. The first one is the high level of under-reported cases in Brazil, both in contagion and number of deaths. Despite being the second country in number of confirmed cases now (almost 700.000), Brazil is not even in top 100 in number of tests per one million citizens. The other aspect is that we initiated a more strict quarantine around 15th April, which reduced the spread ratio. See the graph in Fig. 1 of our reply. So, the combination of both these factors contributed to this discrepancy.

In fact, the total number of infect persons is roughly estimated to be between 5-15 times what is reported (see, for instance, technical notes (in Portuguese) at https://wp.ufpel.edu.br/covid19/artigos-cientificos/). Most recent studies conclude that Brazil has already more than 5 million infected individuals. Regarding the number of deaths, there are recent studies estimating that the actual number can be up to 140% larger. This conclusion is based on the comparison of the number of current deaths due to acute respiratory diseases with the historical average. From this data analysis, it is estimated that at 2nd of May Brazil had already 16.144 deaths caused by Covid-19 instead of the 6.724 confirmed ones See for instance, https://www1.folha.uol.com.br/equilibrioesaude/2020/05/dados-do-governo-indicam-140-a-mais-de-mortes-por-covid-no-pais.shtml (in portuguese). Also, we have expanded the axes limits in Fig. 2 so that it can be easily inferred the performance in the short term (up to April 6). We have added a few sentences in the manuscript mentioning that.

Reply to the Referee C

We thank the Referee for the positive assessment of our paper and for recommending publication. We have discuss all the modifications suggested by the Referee, as shown below.

Referee C: *Reviewer 3: The manuscript is technically solid. The data used in the analysis is available at Johns Hopkins University COVID-19 database. The method used is well known and has been used in similar articles. The article has been published in a preprint version, including the legend that has not been peer-reviewed.*

Our reply: We thank the Referee again for the positive report. Below we provide our replies.

Referee C: *I suggest that the authors should take into account the contact matrix in the analysis, because the present analysis shows the direct effect of the NPI interventions. If the contact matrix are incorporated the authors can include the indirect effects generated from contacts between different age groups.*

Our reply: As mentioned to referee A, although an uniform contact matrix is a crude first approximation, a more nuanced description can be incorporated in the infection rate parameter β (that is age structured in our model) as in $(\beta^{'}C)SI$, explaining why communities with intense contact show a fast contagious rate (larger β). In other words, we can neglect the contact matrix and rely solely on data-driven estimation of the infections rate as an effective measurement of the joint effects (probability of infections when exposed and the contact matrix). Retrieving this contact matrix is certainly very relevant but very difficult to be done on large scale (considering all of Brazil, for instance) and would be better suited for an independent project.

Referee C: *The title of Table IV should be reviewed, since it should mention the case fatality rate, instead of mortality. Table V should specify whether the information on those infected is until the entire population finishes infecting or until a certain date.*

Our reply: We better discuss the results of Table V and VI as suggested by the Referees. In particular, we added a sentence saying that their estimations is based from the initial date up to 150 days ahead, where the disease is modelled to be controlled.

Referee C: *In the results, it would be of interest for health system planning policies that the demand for UTI beds be presented in each scenario. The conclusions are well developed and conform to those presented in the introduction.*

Our reply: We thank the Referee for the positive report once more.
